# Genome data mining of four probiotic *Pediococcus pentosaceus* strains isolated from healthy dairy cows

**DOI:** 10.1016/j.dib.2026.113145

**Published:** 2026-08-06

**Authors:** Fowzia Homa, Kh. Yeashir Arafat, Sabbir Khan, Md. Anamul Kabir Hemal, Ziban Chandra Das, Tofazzal Islam, M. Nazmul Hoque

**Affiliations:** a Molecular Biology and Bioinformatics Laboratory, Department of Gynecology, Obstetrics and Reproductive Health, Gazipur Agricultural University, Gazipur 1706, Bangladesh; b Institute of Biotechnology and Genetic Engineering, Gazipur Agricultural University, Gazipur 1706, Bangladesh

**Keywords:** Genome sequencing, Assembly, Annotations, Phylogeny, Probiotics, Bacteriocin

## Abstract

*Pediococcus pentosaceus* is a Gram-positive lactic acid bacterium known for food fermentation, probiotic properties, and bacteriocin production. This study reports draft contig-level whole genome sequence data of four *P. pentosaceus* strains (MBBL_PB1 – MBBL_PB4) isolated from the milk of healthy crossbred dairy cows. Genomic DNA extracted from these four strains was sequenced with the Illumina MiSeq (2 × 250 bp) platform. The genome assembly was performed with the SPAdes Genome Assembler (v4.2.0). Assembled genome sizes ranged from 1797,199 to 1875,139 bp, with GC contents ranging from 37.0% to 37.5%, genome completeness exceeding 95.5%, and sequencing coverage depths of 203× to 226×. The assemblies consisted of 82 - 143 contigs, with N50 values ranging from 25,907 to 64,606 bp. Genome annotations predicted 1895–1933 genes per genome, including 1831–1867 protein-coding sequences and 53–55 tRNA genes. rRNA operons were present in all genomes, with 5S, 16S, and 23S rRNA genes occurring in variable copy numbers, and the number of complete rRNA operons ranged from 2–3 per strain. CRISPR array analysis revealed two arrays in MBBL_PB1, MBBL_PB2, and MBBL_PB4, and three arrays in MBBL_PB3. Functional annotation predicted genes associated with carbohydrate metabolism and stress response, as well as bacteriocin related Pfam domains, including PF01721, PF04604, PF08129, and PF10439. The genome sequence data of MBBL_PB1, MBBL_PB2, MBBL_PB3, and MBBL_PB4 are deposited in NCBI GenBank under accession numbers JBTWBE000000000, JBTWBD000000000, JBTWBC000000000, and JBTWBB000000000, respectively, with BioProject accession PRJNA1065156.

Specifications Table

**Table:** 

Subject	Biology
Specific subject area	Microbiology, Genomics, Biotechnology
Type of data	Raw and processed genome sequence (Figures and table)
Data collection	The genomic DNA was sequenced with the Illumina MiSeq platform and paired-end (2 × 250 bp) library preparation protocol. The genome was assembled with the SPAdes Genome Assembler (v4.2.0), and annotated using the NCBI Prokaryotic Genome Annotation Pipeline (PGAP) v6.10. Functional annotations with reference to the subsystem features, antibiotic resistance genes, virulence genes, pathogenicity scores, and putative bacteriocin biosynthetic genes were performed using the RAST v2.0, CARD v3.2.4, VFDB v6.0, PathogenFinder v1.1 and BAGEL v4.0 tools, respectively.
Data source location	The four *P. pentosaceus* strains (MBBL_PB1 – MBBL_PB4) reported in this study were isolated from the milk of healthy crossbred dairy cows located at Gazipur (24.09° N, 90.41° E) district of Bangladesh.
Data accessibility	Repository name: NCBI GenBankBioProject: PRJNA1065156BioSamples: SAMN54710466 - SAMN54710469Assembly Accessions: • MBBL_PB1: GCA_054848105.1 • MBBL_PB2: GCA_054848025.1 • MBBL_PB3: GCA_054848035.1 • MBBL_PB4: GCA_054848065.1 Direct URL:https://www.ncbi.nlm.nih.gov/bioproject/PRJNA1065156
Related research article	None

## 1 Value of the Data

• This dataset provides high-quality whole-genome sequences of four dairy-associated *P. pentosaceus* strains, enabling their comparative genomic analyses.
• The genomes harbour four conserved Pfam domains associated with bacteriocin production, providing genomic evidence for putative antimicrobial peptide biosynthetic features.
• These conserved domain signatures can be exploited as genomic markers to screen newly sequenced *P. pentosaceus* strains for putative bacteriocin biosynthetic potential.
• The dataset can be integrated with publicly available *P. pentosaceus* genomes to investigate the distribution, conservation, and evolution of bacteriocin-related genes across ecological niches.
• These genomic resources support future studies on probiotic trait prediction, antimicrobial potential assessment, and the selection of candidate strains for dairy fermentation and animal health applications.

## 2 Background

*P. pentosaceus* is a homofermentative lactic acid bacterium, and is widely distributed in fermented foods, plant-associated environments, and animal microbiomes [[1], [2], [3]]. As a member of the lactic acid bacteria (LAB) group, *P. pentosaceus* contributes to food safety and microbial balance through its key roles in food fermentation, probiotic activity, and antimicrobial compound production [4,5]. Various *P. pentosaceus* strains exhibited anti-inflammatory, antioxidant, anticancer, and lipid-lowering properties, attracting growing interest for applications in functional foods, animal health, and bio-preservation [4,6]. Recent studies have further highlighted their capacity to tolerate environmental stresses, metabolize diverse carbohydrates, and produce bacteriocins with inhibitory activity against foodborne and veterinary pathogens [[7], [8], [9]]. Moreover, microbial genome sequencing has emerged as a powerful tool for the comprehensive characterization of beneficial microbes [7], enabling identification of genes associated with metabolism, stress tolerance, antimicrobial activity, and safety [[10], [11], [12]]. Despite growing interest in LAB genomics, genomic data for *P. pentosaceus* strains isolated from the milk of healthy cows remain limited. Here, we present draft genome sequences of four probiotic *P. pentosaceus* strains (MBBL_PB1–MBBL_PB4) isolated from the milk of healthy dairy cows, to enhance our understanding on their probiotic potential and intra-species genomic diversity.

## 3 Data Description

This study presents the draft genome sequences and associated genomic features of four *P. pentosaceus* strains (MBBL_PB1–MBBL_PB4) isolated from the milk of healthy, mastitis-free crossbred dairy cows across four farms in Gazipur district (24.0° N, 90.4° E), Bangladesh. Whole-genome sequencing (WGS) followed by *in silico* analyses was employed to investigate genome architecture, coding potential, functional subsystems, bacteriocin repertoire, and safety-related genomic features. The raw read counts for MBBL_PB1, MBBL_PB2, MBBL_PB3, and MBBL_PB4 were 1580,442, 1626,292, 1552,596, and 1469,398, respectively. After quality-filtering, the corresponding read counts were 1482,286, 1520,560, 1450,576, and 1363,652. Kraken2 (v2.1.7) taxonomic classification against the Standard database confirmed the taxonomic identity of all four isolates as *P. pentosaceus*, with species-level abundance ranging from 85.12% to 88.74% and overall classification rates exceeding 95%. The assembled genome sizes ranged from 1797,199 bp (MBBL_PB3) to 1875,139 bp (MBBL_PB1), with GC contents ranging from 37.0% to 37.5%, genome completeness exceeding 95.5%, and sequencing coverage depths of 203× to 226×. N50 values ranged from 25,907 to 64,606 bp, and contig numbers ranged from 82 to 143. Genome annotation predicted approximately 1913 genes (range: 1895–1933), of which about 1836 were protein-coding sequences (range: 1831–1867) and 54 were tRNA genes (range: 53–55). rRNA operons were present in all genomes, with 5S, 16S, and 23S rRNA genes occurring in variable copy numbers, and the number of complete rRNA operons ranged from 2–3 per strain. Each genome also harboured three non-coding RNAs and 18–47 pseudogenes. CRISPR array analysis identified two arrays in MBBL_PB1, MBBL_PB2, and MBBL_PB4, and three arrays in MBBL_PB3 (Table 1).

**Table 1:** Genome features of the four probiotic *P. pentosaceus* strains.

Genome feature	*P. pentosaceus* strains			
	MBBL_PB1	MBBL_PB2	MBBL_PB3	MBBL_PB4
GenBank accession	JBTWBE000000000	JBTWBD000000000	JBTWBC000000000	JBTWBB000000000
BioSample accession	SAMN54710466	SAMN54710467	SAMN54710468	SAMN54710469
SRA accession	SRR36871492	SRR36871491	SRR36871490	SRR36871489
Number of reads (Raw)	1580,442	1626,292	1552,596	1469,398
Number of reads (Post-trim)	1482,286	1520,560	1450,576	1363,652
Reads retained (%)	99.56	99.65	99.44	99.52
Genome size (bp)	1875,139	1822,430	1797,199	1825,208
GC (%)	37.5	37.5	37	37.5
Genome completeness (%)	96.5	96.4	96.6	95.7
Genome Coverage (x)	212	226	217	203
N_50_ value (bp)	64,606	29,250	32,123	25,907
Number of contigs	82	136	126	143
Genes (total)	1933	1909	1895	1915
CDSs (total)	1867	1842	1831	1845
Genes (coding)	1820	1812	1813	1815
CDSs (with protein)	1820	1820	1813	1815
Genes (RNA)	66	67	64	70
rRNAs	4, 2, 2 (5S, 16S, 23S)	4, 3, 2 (5S, 16S, 23S)	3, 2, 3 (5S, 16S, 23S)	4, 3, 5 (5S, 16S, 23S)
Complete rRNAs	2 (5S)	2 (5S)	2 (5S)	3 (5S)
Partial rRNAs	2, 2, 2 (5S, 16S, 23S)	2, 3, 2 (5S, 16S, 23S)	1, 2, 3 (5S, 16S, 23S)	1, 3, 5 (5S, 16S, 23S)
tRNAs	55	55	53	55
ncRNAs	03	03	03	03
Pseudo genes (total)	47	30	18	30
CDSs (without protein)	47	30	18	30
Pseudo genes (ambiguous residues)	0 of 47	0 of 30	0 of 18	0 of 30
Pseudo genes (frameshifted)	14 of 47	9 of 30	5 of 18	11 of 30
Pseudo genes (incomplete)	25 of 47	15 of 30	7 of 18	13 of 30
Pseudo genes (internal stop)	13 of 47	8 of 30	7 of 18	8 of 30
Pseudo genes (multiple problems)	5 of 47	2 of 30	1of 18	2 of 30
CRISPR arrays	2	2	3	2
Number of ARGs	1	0	2	0
Number of VFGs	0	0	0	0
Number of subsystems	199	199	202	199
Pathogenicity score (out of 1.0)	0.169	0.167	0.17	0.169
Matched pathogenic families	0	0	0	0
Matched not pathogenic families	127	126	119	124

N.B. Pathogenicity score: 0.8–1.0 → High likelihood of pathogenicity; 0.5–0.8 → Moderate likelihood; < 0.5 → Low likelihood (more likely non-pathogenic or commensal/probiotic).

**Fig. 1:**
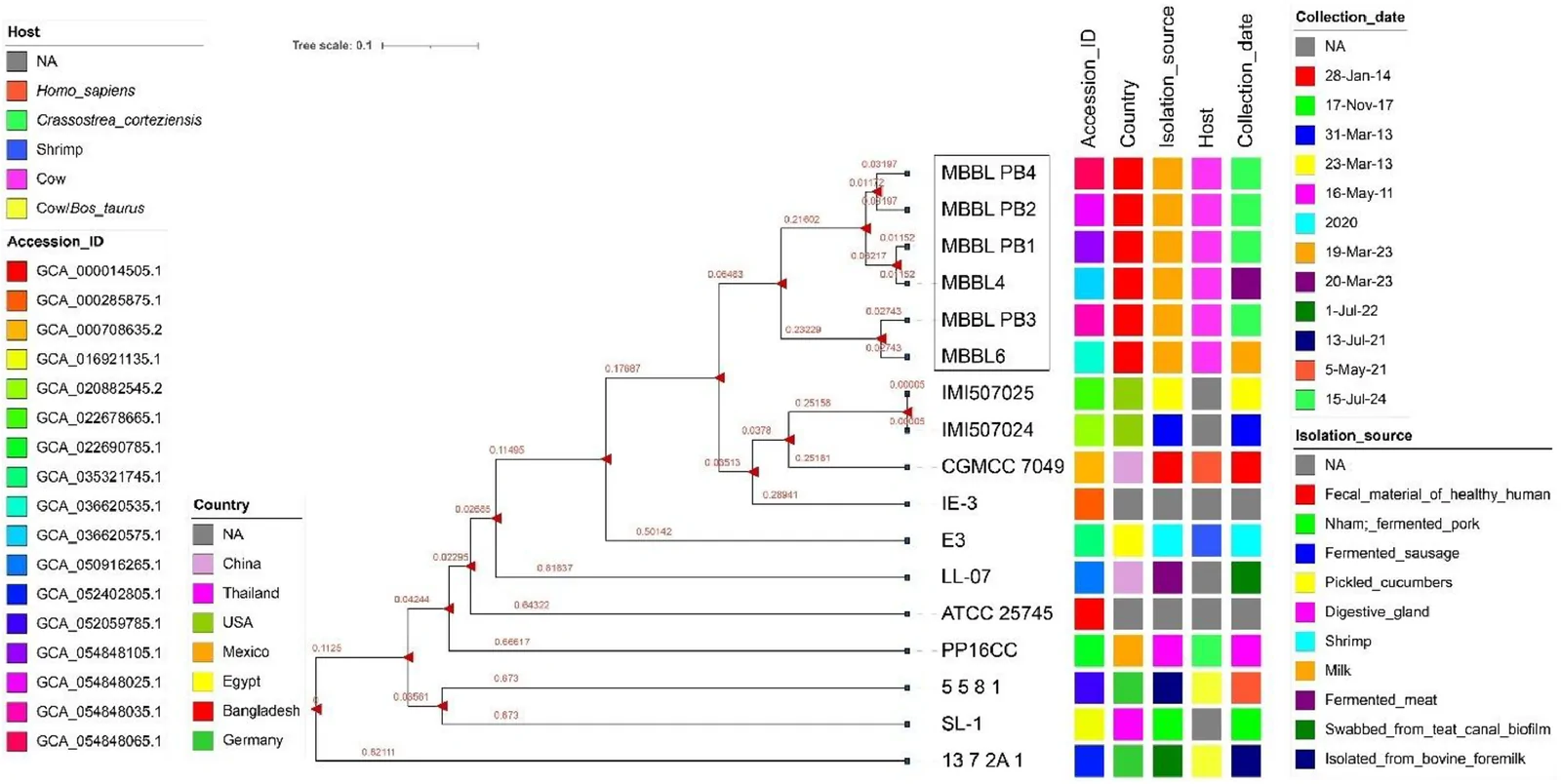
Phylogenetic placement of four probiotic *P. pentosaceus* strains. ANI-based distance dendrogram shows the relationship of four strains isolated in this study (MBBL_PB1–MBBL_PB4, highlighted in black box) with 13 publicly available *P. pentosaceus* genomes from NCBI GenBank. The tree is rooted at the midpoint, and branch lengths reflect ANI-derived distances (1 − ANI/100). The scale bar represents a distance of 0.1. The four study isolates are distributed across two distinct sub-clades within the *P. pentosaceus* clade, indicating genetic heterogeneity. Three strains (MBBL_PB1, MBBL_PB2, MBBL_PB4) form a distinct sub-clade with *P. pentosaceus* MBBL4, whereas MBBL_PB3 clustered with MBBL6. Strains MBBL4 [10] and MBBL6 [4] were previously sequenced by our group from similar dairy sources, provide internal reference points, indicating that the study isolates clustered into two distinct groups within the selected reference dataset.

**Fig. 2:**
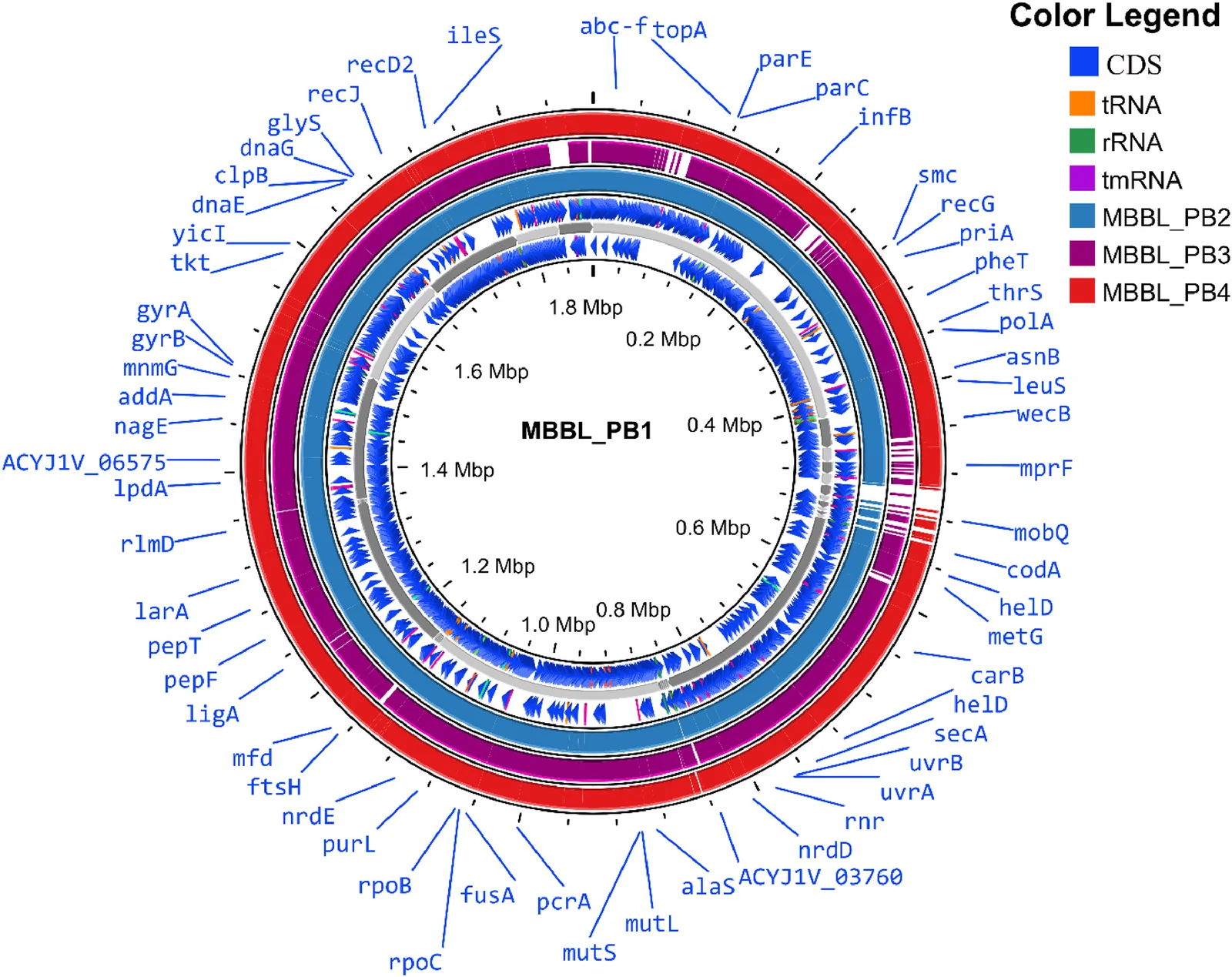
A circular genomic map of *P. pentosaceus* strain MBBL_PB1. The circular representation illustrates the distribution of genomic features. From the innermost to outermost rings: Ring 1: coding DNA sequences (CDSs; orange) Rings 2: transfer RNA (tRNA) genes (red); Ring 3: ribosomal RNA (rRNA) genes (lime green) and Ring 4: and transfer-messenger RNA (tmRNA) genes (purple). The map was generated using the Proksee web server (https://proksee.ca).

**Fig. 3:**
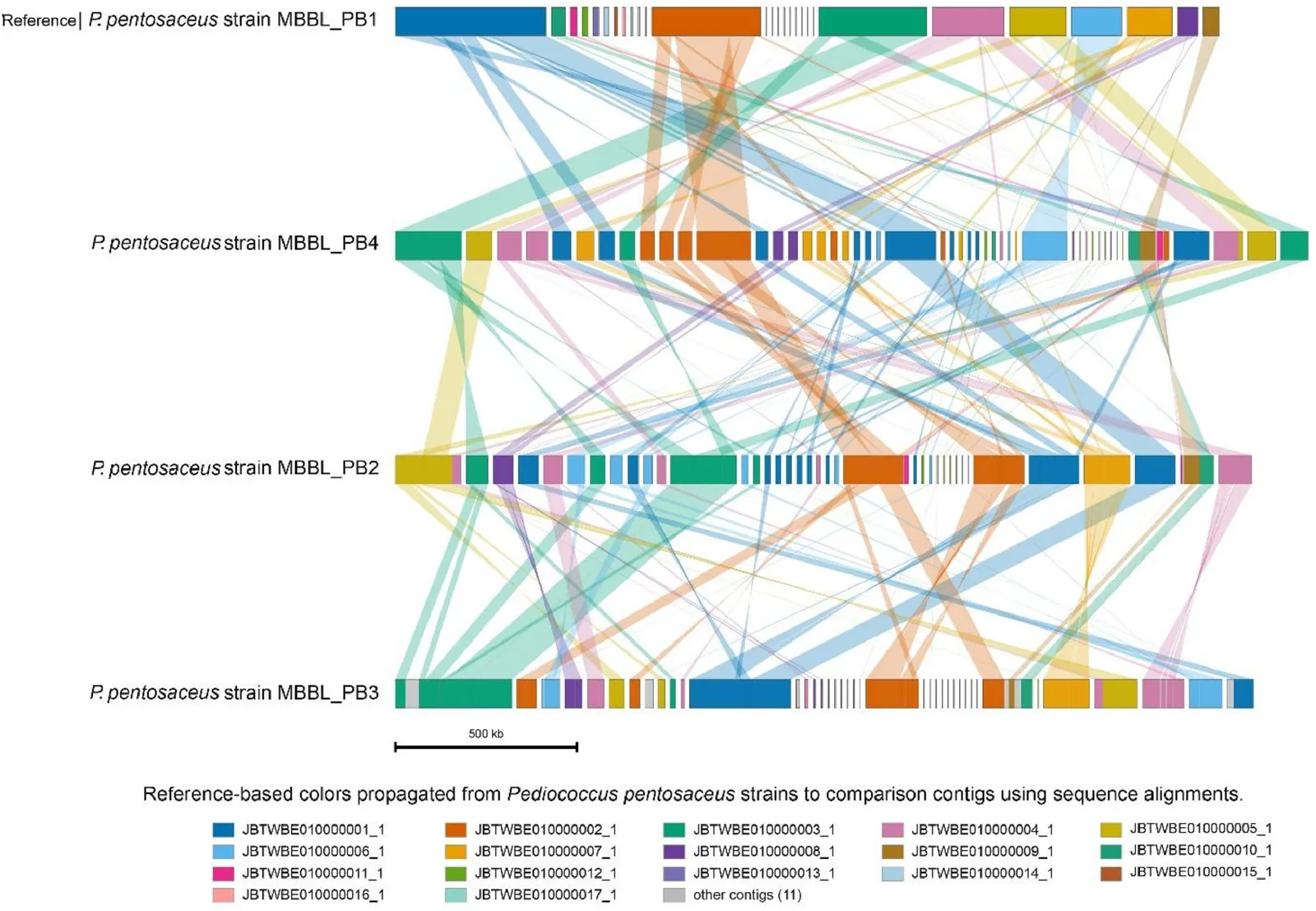
Comparative genome ribbon plots among four *P. pentosaceus* strains. Genome of *P. pentosaceus* MBBL_PB1 was considered as the reference genome for the rest of the three genomes. The plots were generated using genome-loom v0.5.3 implemented in Proksee web server (https://proksee.ca).

Comparative genome analysis revealed a very low abundance of antibiotic resistance genes (ARGs), with only a single ARG detected in MBBL_PB1, two in MBBL_PB3, and an absence of ARGs in both MBBL_PB2 and MBBL_PB4. Furthermore, no virulence factor gene (VFG) was predicted in any strain. Pathogenicity assessment yielded consistently low scores (0.167–0.170), with no matches to pathogenic families, whereas each genome exhibited 119–127 matches to non-pathogenic families. An average nucleotide identity (ANI)-based clustering analysis of the four study strains and 13 reference *P. pentosaceus* genomes (Fig. 1) [2,4,10,[13], [14], [15], [16], [17], [18], [19], [20], [21]] demonstrated that MBBL_PB1, MBBL_PB2, and MBBL_PB4 clustered together in a single sub-clade with the previously characterized probiotic *P. pentosaceus* strain MBBL4 [22], whereas MBBL_PB3 segregated into a distinct sub-clade with another reference probiotic strain, MBBL6 [4]. Despite this sub-clade differentiation, all four isolates were positioned within the core *P. pentosaceus* lineage, corroborating their species-level assignment and close evolutionary relationship to established probiotic strains (Fig. 1). Genome of *P. pentosaceus* MBBL_PB1 was visualized as a circular genome map (generated using the Proksee web server), displaying distinct distribution patterns of CDSs, tRNAs, rRNAs, tmRNAs etc. across the genome (Fig. 2). Comparative genome alignment indicated close genomic synteny and overall structural similarity among the four *P. pentosaceus* isolates (Fig. 3).

**Fig. 4:**
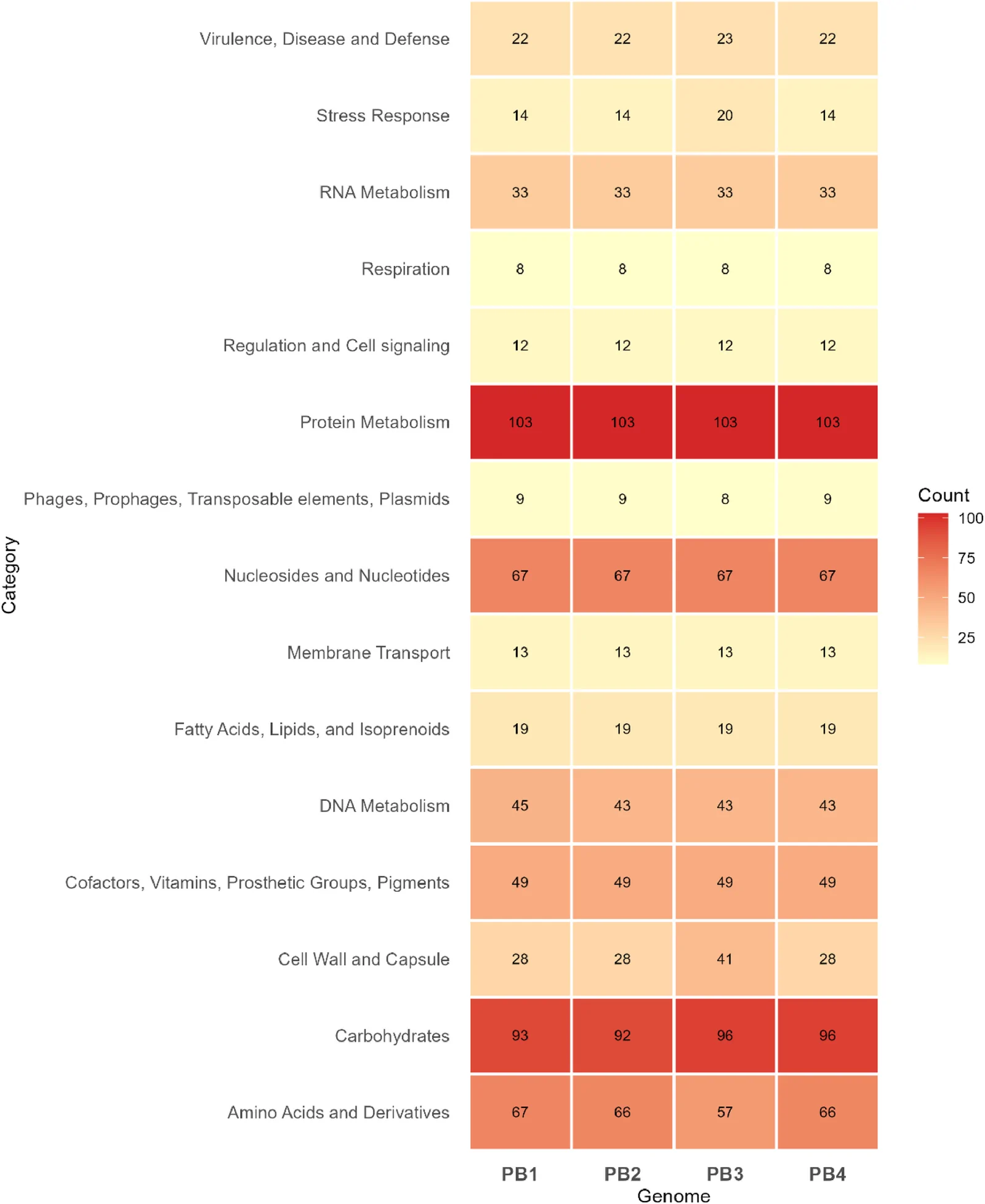
Metabolic functional potentials of the four probiotic *P. pentosaceus* strains**.** Heatmap showing the distribution of SEED subsystem functional categories across the four *P. pentosaceus* genomes (MBBL_PB1 through MBBL_PB4). Each cell displays the number of annotated genes assigned to the corresponding subsystem category in each genome. Cell colour intensity reflects the gene count, ranging from pale yellow (low count, ∼25) to dark red (high count, ≥100), as indicated by the colour scale on the right.

Functional annotation performed with the Rapid Annotations using Subsystems Technology (RAST) tool identified 15 distinct subsystem categories. Protein metabolism was the most represented category (103 genes across all strains), followed by carbohydrate metabolism (92–96 genes) and amino acid biosynthesis (57–67 genes). Nucleosides, nucleotides, cofactors, and vitamins were consistently distributed across all genomes (Fig. 4). Stress response related genes were identified in all genomes, with 20 genes detected in MBBL_PB3 and 14 genes in each of the remaining strains. Bacteriocin screening using BAGEL4 identified conserved Pfam domains in all four genomes: PF01721 (Class II bacteriocin), PF04604 (Type-A lantibiotic), PF08129 (alpha/beta enterocin family), and PF10439 (Class II bacteriocin with double-glycine leader peptide).

## 4 Experimental Design, Materials and Methods

### 4.1 Sample collection and bacterial isolation

Milk samples were collected on 15 July 2024 from four healthy lactating crossbred cows belonging to four small-holding dairy farms (10–20 animals per farm) in Gazipur (24.09° N, 90.41° E), Bangladesh, following proper hygienic procedures. Prior to sampling, udders and teats were visually inspected for any signs of clinical mastitis. Teats were then cleaned using sterile cotton soaked in 70% ethanol and allowed to air dry. The first three to four streams of milk were discarded from each teat to reduce the risk of teat canal contamination. Approximately 2–3 mL of milk was then aseptically collected from each of the four quarters of each cow into a sterile Falcon tube and subsequently pooled to obtain a composite quarter milk sample per animal. Care was taken to avoid contact between the collection vessel and teat ends to minimize environmental contamination. Samples were immediately placed on ice and transported to the laboratory for further analysis. Udder health status was confirmed using the California Mastitis Test (CMT; TechniVet, USA) following standard protocols [23], and only CMT-negative cows were included in the study. The collected samples were properly labelled, maintained at 4 °C in an insulated ice box, and transported to the laboratory for processing within 3–5 h to ensure maximum viability and optimal recovery of bacterial isolates. Isolation of *P. pentosaceus* was performed under aseptic conditions using standard microbiological techniques [4,7,22]. Briefly, 100 µL of each milk sample was inoculated into 5 mL de Man, Rogosa and Sharpe (MRS) broth (HiMedia, India) and incubated aerobically at 37 °C for 24 h for bacterial enrichment. The enriched broth was then streaked onto MRS agar, and incubated at 37 °C for 48 h. Presumptive colonies exhibiting characteristic creamy-white, circular, mucoid morphology were selected and purified by repeated subculturing (3–4 passages) on fresh MRS agar to obtain pure isolates [15,22]. Species-level identification of *P. pentosaceus* was confirmed using the VITEK® 2 automated identification system v9.02 (bioMérieux, USA) and the corresponding VITEK® 2 GP card designed for Gram-positive lactic acid bacteria [10]. Pure colonies grown on MRS agar were suspended in 0.45% saline to achieve a 0.5 McFarland turbidity standard and subsequently inoculated into VITEK 2 system cards. After 3 h of incubation, the system identified the MBBL_PB1, MBBL_PB2, MBBL_PB3, and MBBL_PB4 isolates as *P. pentosaceus* based on their biochemical profiles. Thereafter, the four confirmed isolates were selected for DNA extraction, WGS, and downstream genomic analyses.

### 4.2 DNA extraction, library preparation and sequencing

The isolates were subcultured overnight in MRS broth (HiMedia, India) at 37 °C. Bacterial cells were harvested by centrifugation at 14,000 rpm for 5 min, followed by genomic DNA extraction using the QIAamp DNA Mini Kit (QIAGEN, Germany). DNA purity was assessed using a NanoDrop 2000 spectrophotometer (Thermo Fisher Scientific, USA), with high-quality DNA indicated by A260/280 ratios of approximately 1.8 and A260/230 ratios of approximately 2.0 [24]. DNA concentration was subsequently measured with a Qubit 4.0 fluorometer (Thermo Fisher Scientific, USA) according to the manufacturer’s instructions. Genomic libraries were prepared from 1 ng of DNA using the Nextera DNA Flex Library Preparation Kit (Illumina, San Diego, CA, USA). WGS was then performed on an Illumina MiSeq platform (Illumina, USA) using a 2 × 250 bp paired-end sequencing protocol [25].

### 4.3 Genome assembly and annotation

Raw read quality was evaluated using FastQC v0.12.0 [26]. The reads were then processed with Trimmomatic v0.39.2 [27] to remove Illumina adapter sequences, sequencing artifacts, and phiX control reads. Filtering criteria included a minimum Phred quality score of 30, a minimum read length of 35 bp, and trimming of 15 nucleotides from the 5′ ends of both R1 and R2 reads [28,29]. Taxonomic classification of the high-quality reads (Phred score > 33) was performed using Kraken2 v2.1.7 [30] against the Standard Kraken2 database (2021 release) using default parameters. Thereafter, the reads were assembled using SPAdes v4.2.0 [31] in isolate mode (–isolate), with default parameters. However, contigs shorter than 500 bp were removed using the reformat.sh utility as a standard quality-filtering step to exclude low-confidence and fragmented contigs prior to downstream genomic analyses [32]. Genome annotation was performed using the NCBI Prokaryotic Genome Annotation Pipeline v6.10 [33]. Genome completeness was assessed using CheckM v1.2.4 [34]. Functional subsystem categorization was performed with the RAST v2.0 server [35]. CRISPR arrays were predicted using CRISPRCasFinder v4.2.20 [36]. ARGs were identified using the Comprehensive Antibiotic Resistance Database (CARD) v3.2.4 [37], and VFGs were predicted using the Virulence Factor Database (VFDB) v6.0 [38] bundled in ABRicate (https://github.com/tseemann/abricate) on a Linux command-line platform. Pathogenicity was predicted using the PathogenFinder v1.1 [39].

### 4.4 Phylogenetic reconstruction, circular genome map visualization and bacteriocin prediction

Pairwise ANI values among the four *P. pentosaceus* strains and 13 reference genomes [2,4,10,[13], [14], [15], [16], [17], [18], [19], [20], [21],^40^,^41^] were calculated using FastANI v1.33 [42]. The ANI similarity matrix was converted into a distance matrix (1 − ANI/100), and hierarchical clustering was used to generate an ANI-based distance dendrogram. The resulting dendrogram was visualized with the ggtree package v3.8.0 [43]. A circular genome map visualization was generated from the draft contig-level assembly using the Proksee web server (https://proksee.ca). In addition, comparative genome ribbon plots among four genomes were created uaing genome-loom v0.5.3 implemented in Proksee web server (https://proksee.ca). Putative bacteriocin biosynthetic gene clusters were identified using the BAGEL v4.0 web-based platform [44]. Default parameters were applied for all tools unless otherwise specified.

## Limitations

This study is limited by its small sample size, with only four *P. pentosaceus* strains from a single geographic region (Gazipur, Bangladesh), which limits the generalizability of the findings and may not capture the broader genetic diversity of dairy-associated *P. pentosaceus* populations across different environments or geographic locations. The phylogenetic analysis was based on a limited set of closely related reference genomes rather than a comprehensive collection of publicly available dairy- or host-associated *P. pentosaceus* strains, potentially constraining the scope of comparative genomic interpretations. All functional annotations (e.g., antimicrobial resistance, virulence, CRISPR, bacteriocin production) are derived solely from bioinformatic predictions and lack experimental validation. These results should therefore be interpreted as computational findings requiring future phenotypic confirmation. Importantly, the study involved only non-invasive milk collection from healthy cows under institutional animal welfare approval and did not include animal experimentation. The ethics statement has been clarified to resolve any ambiguity regarding compliance with ARRIVE guidelines in a non-experimental context.

## Ethics Statement

All experimental procedures were reviewed and approved by the Animal Research Ethics Committee (AREC) of Gazipur Agricultural University, Bangladesh (approval no FVMAS/AREC/2023/6679; 16 January 2023). All methods were carried out in accordance with relevant institutional and national guidelines and regulations, and this study is reported in compliance with the ARRIVE guidelines (https://arriveguidelines.org). Furthermore, the authors have read and followed the ethical requirements for publication in *Data in Brief* and confirm that the current work does not involve human subjects, animal experiments, or data collected from social media platforms.

## CRediT Author Statement

**Fowzia Homa:** Sample collection, Investigation, Data curation, Visualization, Writing – original draft preparation. **Kh. Yeashir Arafat:** Sample collection, Investigation, Data curation, Visualization, Writing – original draft preparation. **Sabbir Khan:** Methodology, Data curation, Formal analysis, Visualization. **Md. Anamul Kabir Hemal:** Methodology, Data curation, Formal analysis, Visualization. **Ziban Chandra Das:** Project Administration, Supervision, Funding Acquisition, Validation, Writing – review & editing. **Tofazzal Islam:** Project Administration, Supervision, Validation, Writing – review & editing. **M. Nazmul Hoque:** Conceptualization, Project Administration, Supervision, Funding Acquisition, Methodology, Validation, Writing – review & editing.

## Data Availability

NCBIMining probiotic biogold from dairy animals (Original data).
